# Sex Differences in the Expression of Neuroimmune Molecules in the Spinal Cord of a Mouse Model of Antiretroviral-Induced Neuropathic Pain

**DOI:** 10.3390/biomedicines11030875

**Published:** 2023-03-13

**Authors:** Maryam W. Al-HadlaQ, Willias Masocha

**Affiliations:** 1 Molecular Biology Program, College of Graduate Studies, Kuwait University, Safat 13110, Kuwait; 2 Department of Pharmacology and Therapeutics, College of Pharmacy, Kuwait University, Safat 13110, Kuwait

**Keywords:** antiretroviral drug, ddC, sex differences, neuroinflammation, gene expression, protein expression, microglia, T cells, cytokines, neuropathic pain, mechanical allodynia

## Abstract

Nucleoside reverse transcriptase inhibitors (NRTIs), drugs used to treat HIV infection, can cause neuropathic pain (NP) and neuroinflammation. An NRTI, 2′-3′-dideoxycytidine (ddC), was reported to induce mechanical allodynia and increase proinflammatory cytokines in the brains of female mice. In some models of NP, microglia activation is important for NP pathophysiology in male mice, while T cells are important in female mice. Age-matched female and male mice (BALB/c strain) treated intraperitoneally once daily with ddC for 5 days developed mechanical allodynia. Treatment with ddC increased *Cd11b*, *H2-Aa*, *Cd3e*, *Mapk1*, *Il1b*, *Tnf*, and *Il10* mRNA levels in the spinal cords of female, but not male, mice, whereas there was no alteration found in *Gfap* and *Mapk14* transcripts in both sexes on day 7 after ddC administration. The protein expression of CD11b and phospho-p38 MAPK was significantly increased in the spinal cords of ddC-treated female, but not male, mice, whereas Iba1 protein was elevated in ddC-treated male mice. There was no change in GFAP, CD3e, and phospho-p44/42 MAPK protein levels in both sexes. Thus, changes in neuroimmune cells and molecules in the spinal cords during ddC-induced neuroinflammation were sex-dependent, with female mice being more prone to neuroimmune changes than male mice.

## 1. Introduction

Antiretroviral therapy (ART) is recommended for the treatment, prevention, and management of human immunodeficiency virus (HIV) infection [1]. There are six different classes of antiretroviral drugs that are characterized based on their drug profile and molecular mechanism, which are nucleoside reverse transcriptase inhibitors (NRTIs), nonnucleoside reverse transcriptase inhibitors (NNRTIs), integrase strand transfer inhibitors (INSTIs), protease inhibitors (PIs), chemokine coreceptor antagonists (CCRAs), and fusion inhibitors (FIs) [2]. The recommended initial ART regimens for naïve patients with HIV involve the combination of two NRTIs plus a drug chosen from INSTIs, NNRTIs, or PIs [3]. Although these drugs do not eliminate the virus entirely, they allow for maximal viral suppression [4]. Recently, the European AIDS Clinical Society (EACS) 2019 guidelines recommended a two-drug ART regimen in the treatment of naïve patients involving the combination of two NRTIs: dolutegravir (DTG) and lamivudine (3TC) [5]. It has been observed that NRTIs are mainly associated with peripheral neuropathy (PN) and neuropathic pain (NP) whether used alone or in combination with other drugs [6,7]. It has been shown that symptoms of NP caused by NRTIs ranged from 10–25% for 1 year to over 50% for 2 years after the exposure [8]. However, NP caused by NRTIs can develop in a period of less than a year. One study reported that neuropathy caused by NRTIs peaked in the first 90 days of exposure [6]. In a recent study, zalcitabine (2′,3′-dideoxycytidine, ddC), an NRTI, was found to induce mechanical allodynia and neuroinflammation in the brains of female mice [9].

In the brain, activated microglia induce neuroinflammation through the production and release of several cytokines, chemokines, and neurotoxic compounds [10]. Neurotoxicity in both the central nervous system (CNS) and peripheral nervous system (PNS) seems to be associated with major types of antiretroviral drugs, particularly NRTIs. Amongst the NRTIs, ddC has been used over the years in several animal models in order to understand the pathophysiology or discover treatment targets for ART. Administration of ddC produced mechanical hypersensitivity and allodynia [11], thermal hyperalgesia [12], and reduced conduction velocity in C fiber afferents [13].

Sex is a critical factor in modulating the experience of pain, as males experience pain differently from females and respond differently to specific classes of analgesics [14]. It has been shown that females have a high prevalence of NP than males [15]. Immune cells contribute to sex differences in pain, possibly because of the effects of gonadal hormones (estrogens, progesterone, and testosterone) on them [16]. Sex is an important biologic variable in preclinical research as it was recently considered to be a major predictor of susceptibility to neurological disorders [17]. Microglia, the immune resident cells of the CNS, have a significant role in both sexual differentiation of the brain and the progression of most neurological disorders [18,19]. Sex differences in microglia occur among various regions with respect to both CNS physiology and pathology. It can result from intrinsic factors such as hormonal, environmental, epigenetic modifiers, or X chromosome, which contain a large density of immune-related genes [20]. It has been found that males develop early-onset neurodevelopmental disorders [21], whereas females develop adult-onset neurological disorders [22,23]. The possible reason behind this is because males have more amoeboid microglia and activated innate immune cells in the normal development of sexual differentiation which may alter neuronal function due to microglia over-activation [17]. Recently, studies have revealed that microglia density and phenotype vary between both sexes in many brain areas in early development and aging. Women have higher blood flow compared to men, and the difference in blood flow may contribute to differential microglia density [24]. Microglia proceed from myeloid precursors, which migrate to the CNS from the yolk sack prior to hematopoiesis, at this stage microglia have a role in brain development and physiology, as they are likely to be sex-specific since sex differences occur during early embryonic development [25]. During development, microglia are primarily in an activated state in the healthy neonatal brain and influence neurodevelopmental processes including neurite growth, synaptic pruning, axon guidance, and apoptosis [18].

Preclinical studies using rodent models of NP revealed that microglial cells in the spinal cord are key mediators of peripheral nerve injury (PNI)-induced pain hypersensitivity [26,27,28], yet the role of microglia in pain depends on sex. Inhibiting microglia activation or depleting in microglia terminated pathological pain behavior such as mechanical allodynia in males only, implying that microglia are not essential for pain hypersensitivity in female mice [29]. In contrast, it has been revealed that pain hypersensitivity in female mice depends on T cells, which are essential to driving the non-microglia pathway, as the T cells migrate into the spinal cord after nerve injury [30,31].

The aim of this research was to study whether there are sex-dependent differences in the development of NP and neuroinflammation induced by NRTIs. ddC was used as a model chemical for inducing NP especially, NRTI-induced neuropathy. Specifically, we evaluated the development of mechanical allodynia and the expression of markers of microglia, astrocytes, T cells, signaling molecules, and cytokines in the spinal cords from mice treated with an NRTI.

## 2. Materials and Methods

### 2.1. Animals

Female and male BALB/c mice (8–12 weeks old; 20–25 g) were supplied by the Health Sciences Center (HSC)’s Animal Resource Centre, Kuwait University, Kuwait. The animal experimental work conducted for this study was approved by the Ethical Committee for the Use of Laboratory Animals in Teaching and Research, HSC (Ref: 23/VDR/EC/, Date 5 May 2021). Animal handling followed Directive 2010/63/EU of the European Parliament and of the Council on the protection of animals used for scientific purposes. The mice were kept in rooms with controlled temperatures (23 ± 1 °C), light-dark cycles (lights on at 06:00 am and lights off at 18:00 pm), and had food and water ad libitum. All experiments were performed at the same period (08:00 am and 15:00 pm) to eliminate diurnal variations in response. 

### 2.2. Drug Treatment

Prepared freshly in normal saline was 2′,3′-dideoxycytidine (ddC, zalcitabine) (Sigma Aldrich, St. Louis, MO, USA) before administration intraperitoneally (i.p.) to mice at a dose of 25 mg/kg in a volume of 10 mL/kg daily for 5 consecutive days to induce neuroinflammation, similar to what was reported previously [9]. Control animals received the vehicle (normal saline) in a volume of 10 mL/kg daily for 5 consecutive days.

Previous treatment regimens used single or two doses of ddC to induce mechanical allodynia in mice and rats [32,33,34] and multiple doses in chronic regimens lasting 3 weeks [35,36]. The mechanical allodynia induced by the single dose regimen was from day 3 and persisted for up to 21 days at the termination of the experiment [34]. In a previous study, we used a single dose of ddC similar to Sanna et al. [34], and we observed that ddC treatment induced mechanical allodynia in mice on day 2 after treatment causing about a 50% reduction in withdrawal threshold compared to before treatment with ddC [33]. The mechanical allodynia induced by the 2-dose regimen started one day after beginning the administration and persisted up to about 20 days at the termination of the experiment [32]. A more chronic model has been characterized by Wallace et al. [35] in rats (ddC was injected intraperitoneally three times per week for 3 weeks), and recently used in mice [36]. However, even in these chronic models, the mechanical allodynia was observed as early as 2 days after ddC injection [36], suggesting that the mechanical allodynia to ddC starts much early and possibly the changes associated with it. This allows researchers to study early events or late events associated with antiretroviral-induced NP. We have used a 5-day treatment regimen with ddC with the aim of evaluating the effects of multiple dosing on the development of mechanical allodynia within the first week of treatment. The 5-day treatment induced mechanical allodynia in a previous study i.e., at day 7 post-first ddC injection caused a 67% reduction in withdrawal threshold compared to baseline values [9].

### 2.3. Assessment of Mechanical Allodynia

Mechanical allodynia was assessed in mice using the dynamic plantar aesthesiometer (DPA) (Ugo Basile, Italy), as previously reported [33]. Each mouse was left to habituate for about 60–90 min inside a Perspex enclosure on top of a perforated metal platform. The DPA’s touch stimulator unit, which moves below the perforated platform, has a 0.5 mm diameter metal filament that was directly positioned underneath the hind paw and when a start key on the touch stimulator unit was pressed, the metal filament moved upwards to touch the paw skin and increased force linearly (0.25 g/s). The moment the mice withdrew their paw due to the mechanical stimuli, the force at the time of paw withdrawal was detected and recorded automatically by the DPA. A cut-off force of 5 g, or 20 s, was used. For each mouse, at least four different readings were taken. 

### 2.4. Animal Sacrifice and Tissue Isolation

Mice were anesthetized with halothane and sacrificed by decapitation on the 7th day after the first administration of ddC. The spinal cord was extracted as described previously [37]. Seventy percent ethanol was sprinkled onto the fur to reduce the fur attaching to the dissection instruments and spinal cord. Using forceps and a pair of scissors, the skin was cut along the spinal column in a distal-proximal direction, and isolation of the spinal column was carried out by cutting the right and left sides along the spinal column above the pelvic bone. The spinal cord was cut with a pair of scissors far away from the pelvic bone. The spinal column was straightened by applying an index finger to the bent part of the column. A pipette tip (100 µL) was inserted in the spinal cavity at the distal-most end of the spinal column. Steady pressure was applied to extrude the spinal cord into a Petri dish, filled with sterile phosphate-buffered saline (PBS). The spinal cord was picked by forceps from the Petri dish immediately and immersed in liquid nitrogen and stored at −80 °C for subsequent RNA and protein extraction.

### 2.5. RNA Extraction and cDNA Synthesis

Total RNA was extracted from a fresh frozen spinal cord, which was sonicated in 1.5 mL of lysis buffer with 15 µL of 2-mercaptoethanol on ice, using the RNeasy Kit (Qiagen GmbH, Hilden, Germany) as previously described [38]. Quantification of the RNA sample was carried out using NanoDrop 2000 spectrophotometer (Thermo Scientific, Wilmington, DE, USA), and eventually the sample was kept at −80 °C. The RNA went through DNase treatment before cDNA synthesis using a superscript II RT kit (Invitrogen, Carlsbad, CA, USA) as previously described [38]. The cDNA was stored at −20 °C until used for RT-PCR. 

### 2.6. Real Time-Polymerase Chain Reaction (RT-PCR) 

The mRNA of microglia markers (*Itgam* [*Cd11b*] and *H2-Aa*), astrocytes marker (*Gfap*), a marker for T cells (*Cd3e*), signaling molecules (*Mapk14* [*p38a*] and *Mapk1* [*Erk2*]), and cytokines (*Tnf*, *Il1b* and *Il10*) was quantified in the spinal cord of control vehicle-treated and ddC-treated mice by RT-PCR using the QuantStudio™ 7 Flex Real-Time PCR System (Applied Biosystems, Carlsbad, CA, USA) as previously described [38]. The gene expression of each sample was normalized to the housekeeping gene *Ppia* (cyclophilin). The sequences of oligonucleotide primers that were used, listed in Table 1, were extracted from PrimerBank, or previous publications from our laboratory [9,39,40,41,42], and checked for specificity by BLAST in the GenBank database and/or ordered from (Invitrogen by Life of Technologies).

The 2^−ΔΔCt^ method was used to calculate the relative amount of target gene mRNA in the spinal cord [43]. The fractional cycle number at which the amount of amplified target crosses the set threshold i.e., the threshold cycle (Ct) (i.e., a level set above the background level) was used to calculate the relative gene expression in the experimental and control samples, using housekeeping gene as the normalizer. The following equations were used:

1. ΔCt = Ct target gene—Ct housekeeping gene. The housekeeping gene *Ppia* was used to normalize the number of transcripts of individual animal samples (ΔCt; n = 6 to 8 per group).
2. ΔΔCt = ΔCt of experimental animals—Average of ΔCt of control animals.
3. The fold change in the target genes = 2^−ΔΔCT^. These values were then used to calculate the mean ± standard error of the mean (SEM) or median and interquartile range of the relative expression of the target gene mRNA in the spinal cord of vehicle and ddC-treated mice.

### 2.7. WesTM Capillary-Based Protein Electrophoresis

**Figure 1:**
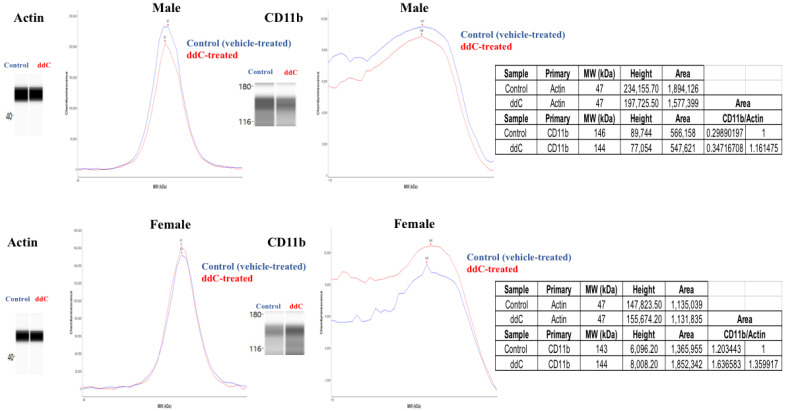
Electropherograms of peak areas for CD11b and Actin in the spinal cord from control-vehicle and ddC-treated female and male mice. The protein expression was measured using the WesTM capillary-based protein electrophoresis. The ratio between the area of the elecropherogram of the protein of interest and the area of β-actin was calculated and normalized to the control group.

The protein expression of microglia markers (CD11b and Iba1), astrocytes marker (GFAP), a marker for T cells (CD3e), and signaling molecules (phospho-p38 MAPK, phospho-p44/42 MAPK), in addition to the housekeeping protein ß-actin, was measured using the automated WesTM capillary-based protein electrophoresis (ProteinSimple, San Jose, CA, USA). EZ Standard Pack 1 containing DDT, 5× fluorescent mix, Biotinylated Ladder (12–230 kDa), Streptavidin-HRP, Luminol, Peroxide, antibody diluent 2, and secondary anti-rabbit antibody was supplied within the kit (ProteinSimple), and used following the manufacturer’s instructions and as previously described [9]. Spinal cord homogenate was diluted to 1 mg/mL using 0.1× buffer, which was originally diluted from 10× buffer. Four parts of the sample were mixed with one part of a 5× fluorescent mix, followed by denaturation at 95 °C for 5 min. The primary antibodies for rabbit anti-CD11b (Abcam, Cambridge, UK), rabbit anti-Iba1 (Boster Bio, Pleasanton, CA, USA), rabbit anti-GFAP (Boster Bio, Pleasanton, CA, USA), rabbit anti-CD3e (MyBioSource, Vancouver, BC, Canada), rabbit anti-phospho-p38 MAPK (Cell Signaling, Danvers, MA, USA), rabbit anti-phospho-p44/42 MAPK (Cell Signaling, Danvers, MA, USA), and rabbit anti-ß actin (Sigma Aldrich, St. Louis, MO, USA) were diluted in a ratio of 1:50 using antibody diluent 2. After loading the samples and the antibodies in the wells, the plate was centrifuged at 2500 rpm for 5 min at room temperature. Then, the plate and a 25-Capillary cartridge were loaded into the Wes instrument, which is automated for the whole process of electrophoresis and immunodetection of the proteins. At the end of the run, the data generated were analyzed using the Compass for Simple Western software (ProteinSimple). The ratio between the peak areas, which represent the signal intensity of the immuno-detected proteins, of the resulting electropherograms for the protein of interest and the housekeeping protein β-actin, was calculated (Figure 1), as described previously [9,42]. Data analysis was performed using Apache OpenOffice Calc and GraphPad Prism.

### 2.8. Statistical Analysis

Data were analyzed using the GraphPad Prism software (version 9.00; GraphPad Software Inc., San Diego, CA, USA). The results were tested for normality using the D’Agostino-Pearson normality test and subsequently analyzed by appropriate statistical tests. An unpaired student’s *t*-test was used to compare responses between different treatment groups if the data were normally distributed. The Mann-Whitney U test was used when the data followed non-normal distribution. Two-way repeated measures ANOVA followed by Sidak’s multiple comparisons test were used to compare the withdrawal threshold between ddC-treated and control vehicle-treated animals at baseline and day 7 after ddC administration. The differences were considered significant when *p* < 0.05. The results were presented as mean ± SEM for normally distributed data or median and interquartile range for skewed data. 

## 3. Results

### 3.1. Effects of ddC on Withdrawal Threshold to Mechanical Stimulation

**Figure 2:**
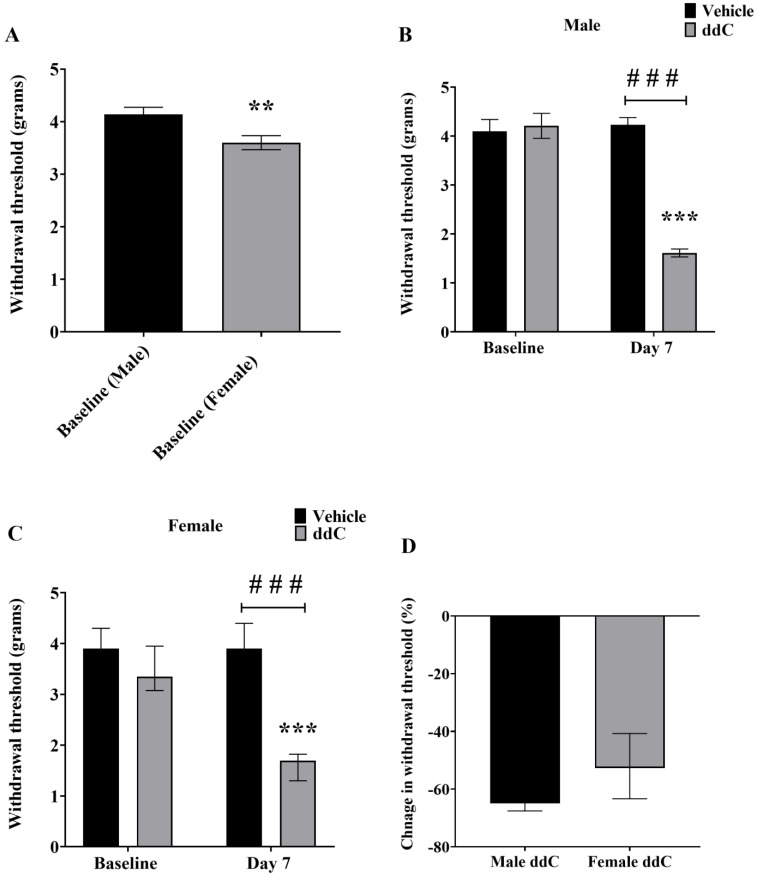
Effect of ddC on mechanical sensitivity of both male and female BALB/c mice. (**A**) Withdrawal threshold to mechanical stimuli of both male and female mice before ddC treatment (baseline). Each bar represents the mean ± SEM obtained from twenty to twenty-seven mice. ** *p* < 0.01 compared to male mice (Unpaired Student’s *t*-test). (**B**) Withdrawal threshold to mechanical stimuli of male mice before (baseline) and at day 7 post ddC (25 mg/kg) injection. Each bar represents the mean ± SEM obtained from eight to nine mice. *** *p* < 0.001 compared to baseline values for ddC-treated male mice (Unpaired Student’s *t*-test). ### *p* < 0.001 at day 7 post-ddC injection compared to control vehicle-treated male mice (two-way repeated measures ANOVA followed by Sidak’s multiple comparisons test). (**C**) Withdrawal threshold to mechanical stimuli of female mice before (baseline) and at day 7 post ddC (25 mg/kg) injection. Each bar represents the median and interquartile range obtained from six to seven mice. *** *p* < 0.001 compared to baseline values for ddC-treated female mice (Mann-Whitney U test). ### *p* < 0.001 at day 7 post-ddC injection compared to control vehicle-treated female mice (two-way repeated measures ANOVA followed by Sidak’s multiple comparisons test). (**D**) Change in withdrawal threshold (%) to mechanical stimuli at day 7 for ddC-treated male and female mice. Each bar represents the median and interquartile range obtained from six to eight mice. No statistically significant differences between the groups were detected (Mann-Whitney U test).

There was a significant difference in withdrawal threshold to mechanical stimuli between male and female mice before ddC administration (baseline) i.e., male mice had a higher withdrawal threshold than female mice (male: 4.141 ± 0.132 g, female: 3.600 ± 0.133 g, *p* = 0.007; Figure 2A). Treatment with ddC (25 mg/kg) for 5 consecutive days significantly reduced the withdrawal threshold of both male and female mice to mechanical stimuli on the 7th day after the first ddC injection compared to baseline values (male: 1.613 ± 0.081 g compared to 4.211 ± 0.255 g, *p* = 0.0001, female: 1.700 (1.300–1.825) compared to 3.350 (3.075–3.950), *p* = 0.0022, respectively), and to control vehicle-treated mice (male: 1.613 ± 0.081 g compared to 4.233 ± 0.146 g, *p* = 0.0001, female: 1.617 ± 0.108 g compared to 3.729 ± 0.262 g, *p* = 0.0001, respectively; Figure 2B,C). Both ddC-treated male mice and ddC-treated female mice developed mechanical allodynia to the same extent i.e., there were no significant differences between the groups (*p* > 0.05; Figure 2D). 

### 3.2. Effect of ddC Treatment on the Gene and Protein Expression of Glial Cells and T Cells Markers in the Spinal Cord of Male and Female Mice

**Figure 3:**
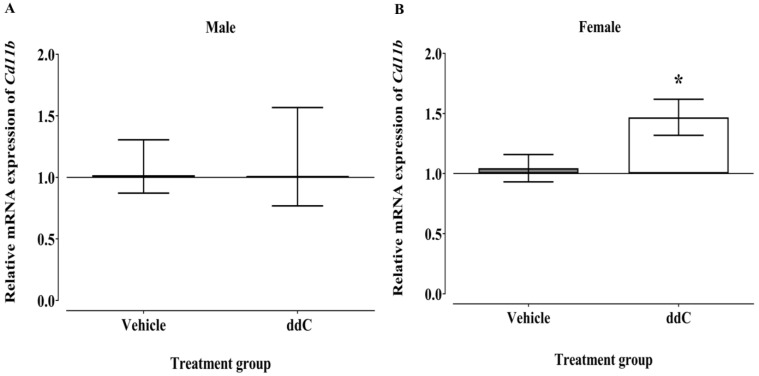
Effect of ddC on the relative mRNA expression of *Cd11b* in spinal cords of (**A**) male and (**B**) female BALB/c mice on the 7th day after first drug treatment. (**A**) Each bar shows the median and interquartile range obtained from eight mice. No differences were detected (Mann-Whitney U test). (**B**) Each bar shows the mean ± SEM obtained from eight mice. * *p* < 0.05 compared to control vehicle-treated female mice (Unpaired Student’s *t*-test).

The mRNA expression of *Cd11b* in the spinal cords of male mice was not significantly affected by ddC treatment compared to vehicle treatment (*p* > 0.05; Figure 3A). In contrast, the expression of *Cd11b* was significantly upregulated in the spinal cords of ddC-treated female mice compared to control vehicle-treated female mice (*p* < 0.05; Figure 3B).

**Figure 4:**
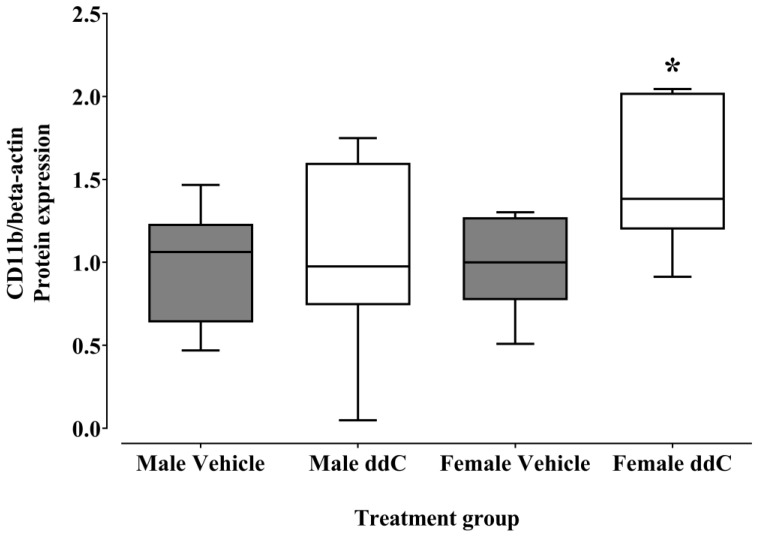
Effect of ddC on protein expression of CD11b in spinal cords of male and female BALB/c mice on the 7th day after first drug treatment. Each box and whisker plot was obtained from seven mice. * *p* < 0.05 compared to control vehicle-treated female mice. Mann-Whitney U test was used to analyze the significance of the results.

Treatment with ddC significantly increased the protein levels of CD11b in the spinal cords of female mice compared to treatment with a vehicle (*p* < 0.05; Figure 4). However, the protein levels of CD11b in the spinal cords of male mice were not affected by ddC treatment compared to vehicle treatment (*p >* 0.05; Figure 4).

**Figure 5:**
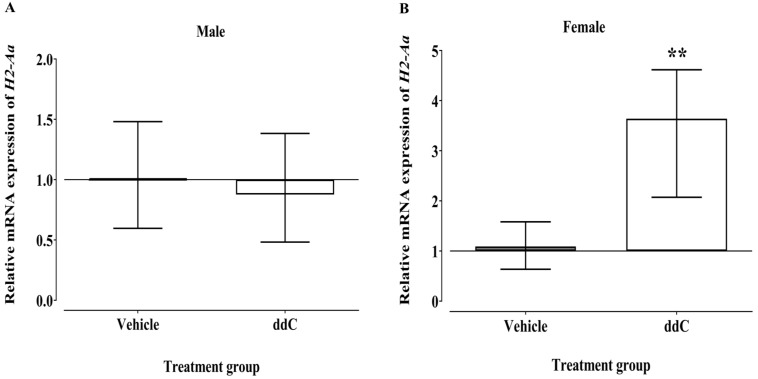
Effect of ddC on the relative mRNA expression of *H2-Aa* in spinal cords of (**A**) male and (**B**) female BALB/c mice on the 7th day after first drug treatment. Each bar shows the median and Interquartile range obtained from seven to eight mice. ** *p* < 0.01 compared to control vehicle-treated female mice (Mann-Whitney U test).

The relative mRNA expression of the MHC-II gene, *H2-Aa* in the spinal cords of male mice was not significantly affected by ddC treatment compared to vehicle treatment (*p* > 0.05; Figure 5A). While the transcript levels of *H2-Aa* were significantly increased in the spinal cords of ddC-treated female mice compared to control vehicle-treated female mice (*p* < 0.05; Figure 5B). 

**Figure 6:**
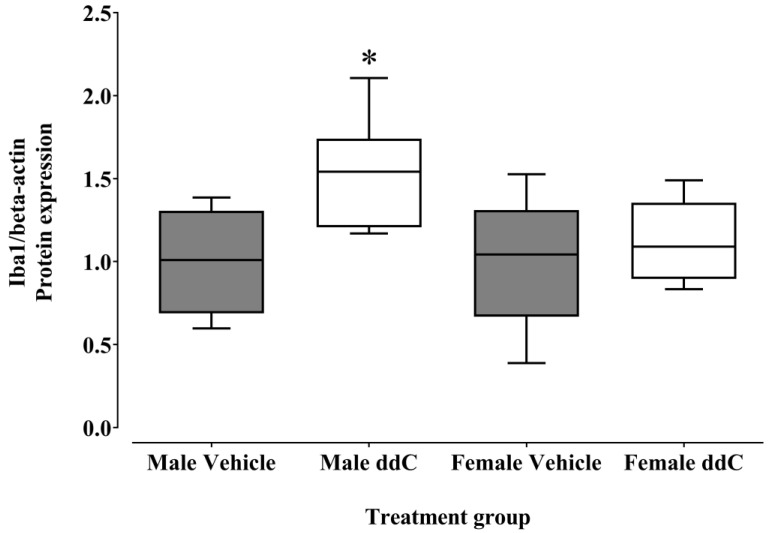
Effect of ddC on protein expression of Iba1 in spinal cords of male and female BALB/c mice on the 7th day after first drug treatment. Each box and whisker plot was obtained from six to seven mice. * *p* < 0.05 compared to control vehicle-treated male mice. Mann-Whitney U test was used to analyze the significance of the results.

There was no change in the protein levels of Iba1 in the spinal cords of ddC-treated female mice compared to control vehicle-treated female mice (*p* > 0.05; Figure 6). In contrast, the protein levels of Iba1 were significantly upregulated in the spinal cords of ddC-treated male mice compared to control vehicle-treated male mice (*p* < 0.05; Figure 6).

**Figure 7:**
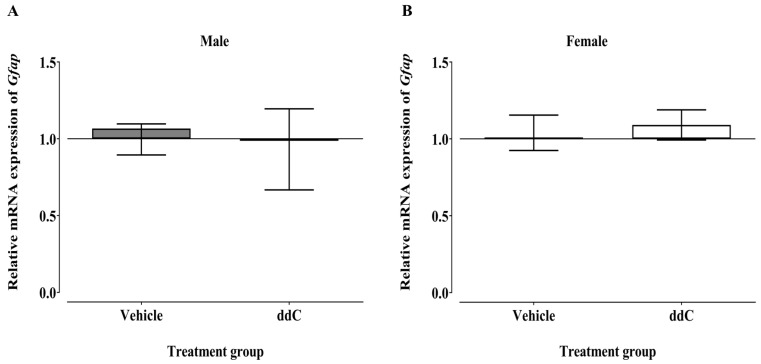
Effect of ddC on the relative mRNA expression of *Gfap* in spinal cords of (**A**) male and (**B**) female BALB/c mice on the 7th day after first drug treatment. (**A**) Each bar shows the median and interquartile range obtained from seven to eight mice (Mann-Whitney U test). (**B**) Each bar shows the mean ± SEM obtained from eight mice (Unpaired Student’s *t*-test). No statistically significant differences between the groups were detected.

Treatment with ddC had no significant effect on the mRNA expression of the astrocytes marker (*Gfap*) in the spinal cords compared to treatment with vehicle in both male and female mice (*p* > 0.05; Figure 7).

**Figure 8:**
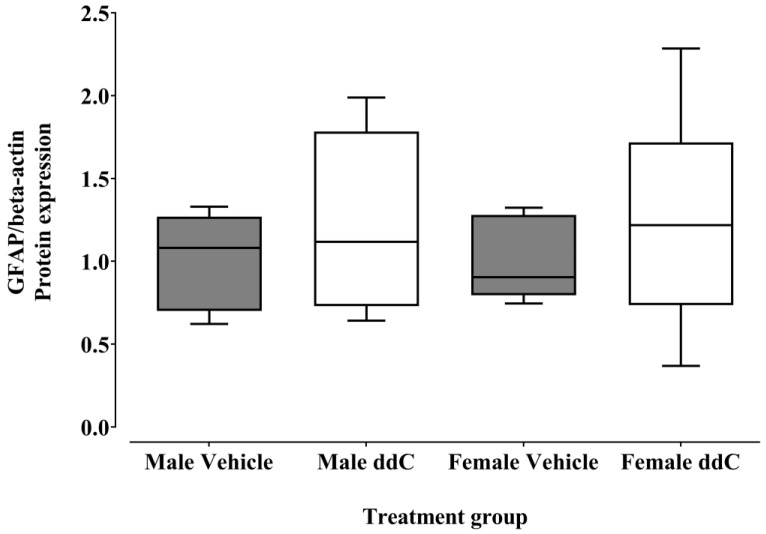
Effect of ddC on protein expression of GFAP in spinal cords of male and female BALB/c mice on the 7th day after first drug treatment. Each box and whisker plot was obtained from six to eight mice. No statistically significant differences between the groups were detected (Unpaired Student’s *t*-test [male] and Mann-Whitney U test [female]).

The administration of ddC did not have a significant effect on the protein levels of GFAP in the spinal cords compared to treatment with a vehicle in both male and female mice (*p* > 0.05; Figure 8).

**Figure 9:**
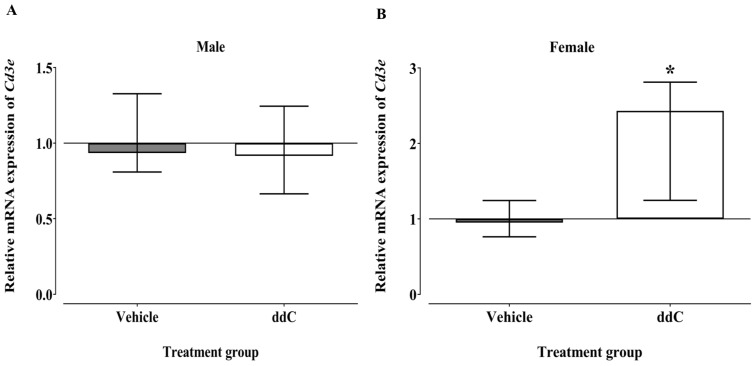
Effect of ddC on the relative mRNA expression of *Cd3e* in spinal cords of (**A**) male and (**B**) female BALB/c mice on the 7th day after first drug treatment. Each bar shows the median and interquartile range obtained from seven mice. * *p* < 0.05 compared to control vehicle-treated female mice (Mann-Whitney U test).

The mRNA expression of the T cells marker (*Cd3e*) in the spinal cords of male mice was not significantly affected by ddC treatment compared to vehicle treatment (*p* > 0.05; Figure 9A). Whereas the mRNA expression *Cd3e* was significantly upregulated in the spinal cords of ddC-treated female mice in comparison with control vehicle-treated female mice (*p* < 0.05; Figure 9B).

**Figure 10:**
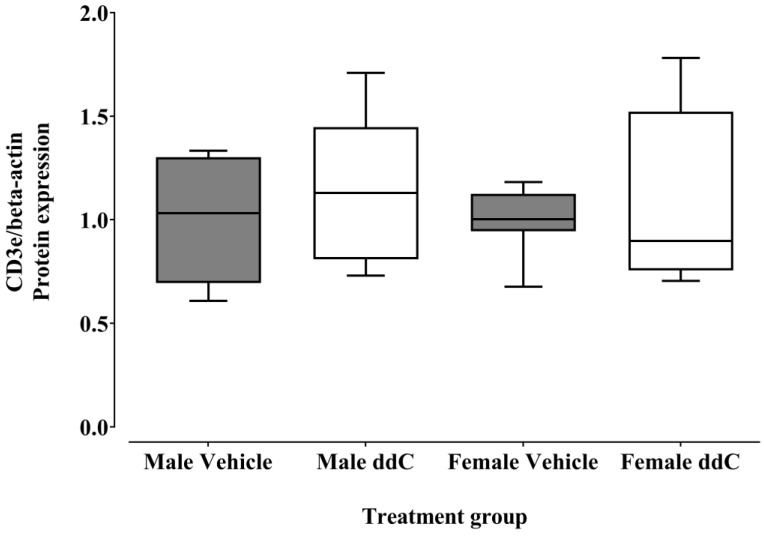
Effect of ddC on protein expression of CD3e in spinal cords of male and female BALB/c mice on the 7th day after first drug treatment. Each box and whisker plot was obtained from eight mice. No statistically significant differences between the groups were detected (Unpaired Student’s *t*-test).

Treatment with ddC did not have a significant effect on the protein levels of CD3e in the spinal cords compared to treatment with a vehicle in both male and female mice (*p* > 0.05; Figure 10).

### 3.3. Effect of ddC Treatment on Gene and Protein Expression of Signaling Molecules ERK1/2 and p38 MAPK in the Spinal Cord of Male and Female Mice

**Figure 11:**
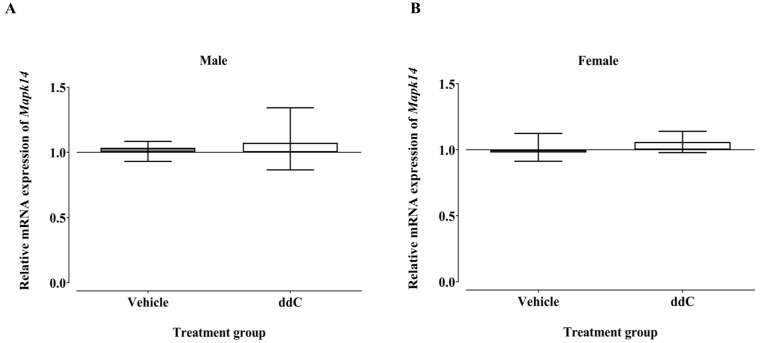
Effect of ddC on the relative mRNA expression of *Mapk14* in spinal cords of (**A**) male and (**B**) female BALB/c strain mice on the 7th day after first drug treatment. (**A**) Each bar shows the median and interquartile range obtained from seven to eight mice (Mann-Whitney U test). (**B**) Each bar shows the mean ± SEM obtained from eight mice (Unpaired Student’s *t*-test). No statistically significant differences between the groups were detected.

There was no significant effect on the mRNA expression of *Mapk14 (p38-α)* in the spinal cords of ddC-treated groups compared to control vehicle-treated groups in both male and female mice (*p* > 0.05; Figure 11A and Figure 11B, respectively). 

**Figure 12:**
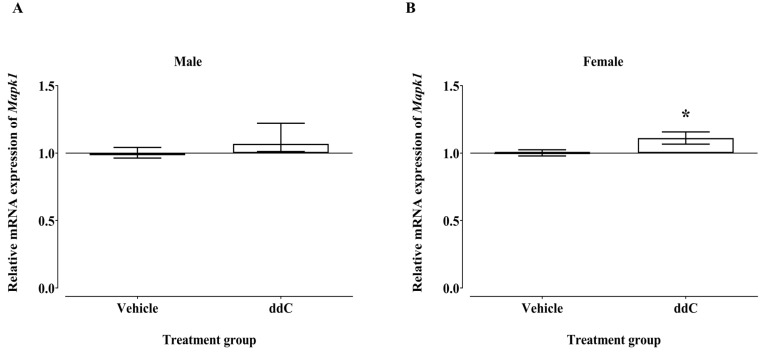
Effect of ddC on the relative mRNA expression of *Mapk1* in spinal cords of (**A**) male and (**B**) female BALB/c strain mice on the 7th day after first drug treatment. (**A**) Each bar shows the median and interquartile range obtained from seven to eight mice (Mann-Whitney U test). (**B**) Each bar shows the mean ± SEM obtained from eight mice. * *p* < 0.05 compared to control-vehicle-treated female mice (Unpaired Student’s *t*-test).

Treatment with ddC did not significantly affect the levels of *Mapk1* (*Erk2*) transcripts in male mice compared to treatment with vehicle (*p* > 0.05; Figure 12A). However, treatment with ddC significantly elevated the transcript levels of *Mapk1* in the spinal cords of female mice compared to treatment with a vehicle (*p* < 0.05; Figure 12B). 

**Figure 13:**
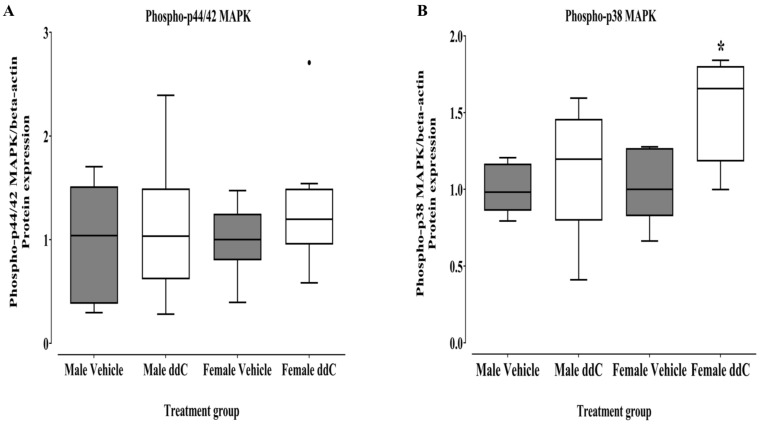
Effect of ddC on protein expression of phospho-p44/42 MAPK (**A**) and phospho-p38 MAPK (**B**) in spinal cords of male and female BALB/c mice on the 7th day after first drug treatment. Each box and whisker plot was obtained from six to eight mice. * *p* < 0.05 compared to control vehicle-treated female mice (Mann-Whitney U test) and (Unpaired Student’s *t*-test) for male phospho-p44/42 MAPK.

The protein levels of phospho-p44/42 MAPK in the spinal cords were not significantly affected by ddC treatment compared to treatment with a vehicle in both male and female mice (*p* > 0.05; Figure 13A). 

There was a significant increase in the protein levels of phospho-p38 MAPK in ddC-treated female mice compared to control vehicle-treated female mice (*p* < 0.05; Figure 13B), but ddC treatment did not significantly affect male mice compared to vehicle treatment (*p* > 0.05; Figure 13B).

### 3.4. Effect of ddC Treatment on Gene Expression of Pro-Inflammatory and Anti-Inflammatory Molecules (Il1b, Tnf and Il10) in the Spinal Cord of Male and Female Mice

**Figure 14:**
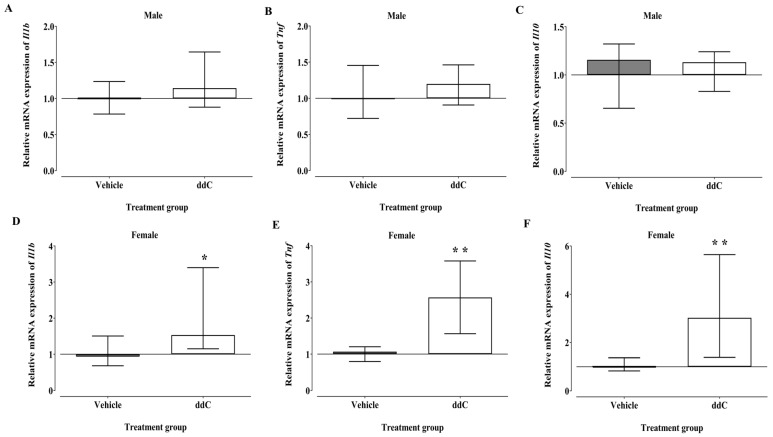
Effect of ddC on the relative mRNA expression of (**A**,**D**) *Il1b* (**B**,**E**) *Tnf* and (**C**,**F**) *Il10* in spinal cords of male and female BALB/c mice on the 7th day after first drug treatment. Each bar shows the median and interquartile range obtained from six to seven mice. * *p* < 0.05, ** *p* < 0.01 compared to control-vehicle-treated female mice (Mann-Whitney U test). This section may be divided into subheadings. It should provide a concise and precise description of the experimental results, their interpretation, as well as the experimental conclusions that can be drawn.

The mRNA expression of *Il1b*, *Tnf*, and *Il10* in the spinal cords of male mice was not significantly affected by ddC treatment compared to treatment with vehicle (*p* > 0.05; Figure 14A, Figure 14B, and Figure 14C, respectively). In contrast, the mRNA expression of pro-inflammatory molecules (*Il1b* and *Tnf*) and anti-inflammatory molecules (*Il10*) was significantly upregulated in the spinal cords of ddC-treated female mice compared to control vehicle-treated female mice (*p* < 0.05; Figure 14D, Figure 14E and Figure 14F, respectively). 

## 4. Discussion

This study revealed sex-dependent differences in the expression of neuroimmune cells and molecules in the spinal cords of BALB/c mice treated with an NRTI, ddC. Naïve female mice were more sensitive to mechanical stimuli than male mice. However, treatment with ddC induced mechanical allodynia equally in both male and female mice. ddC-treated female mice had higher transcripts and protein levels of neuroimmune cells and molecules involved in neuroinflammation in the spinal cords than control vehicle-treated female mice, while there were no significant changes in male mice after ddC treatment, except for Iba1 protein, a microglia marker. The mRNA expression of microglia markers *Cd11b* and *H2-Aa* was significantly upregulated in the spinal cords of female, but not male, mice treated with ddC. The protein expression of microglia marker CD11b was significantly upregulated in the spinal cords of female, but not male, mice, while another microglia marker Iba1 was upregulated in male, but not female, mice treated with ddC. There was no change in the transcript or protein levels of the astrocytes marker GFAP in both male and female mice after ddC treatment. The relative expression of mRNA of the T cells marker *Cd3e* was significantly upregulated in female, but not male, mice treated with ddC, whereas the protein expression of CD3e was not affected by ddC in both sexes. The transcript levels of the signaling molecule *Mapk1* (*Erk2*) were significantly upregulated in ddC-treated female mice, but the male mice were not significantly affected by ddC treatment. The transcript levels of *Mapk14* (*p38-α)* were not affected by ddC treatment in both male and female mice. The levels of phospho-p38 MAPK were significantly upregulated in ddC-treated female mice, whereas the levels of phospho-p44/42 MAPK were not altered. There were no changes in the levels of phospho-p38 MAPK and phospho-p44/42 MAPK in ddC-treated male mice. The mRNA expression of the pro-inflammatory molecules (*Tnf* and *Il1b*) and anti-inflammatory molecule (*Il10*) was significantly elevated by ddC in female, but not male, mice. These findings suggest that the changes in the expression of microglia markers, T cells marker, and neuroimmune molecules during antiretroviral drug-induced NP are sex-dependent and more complex than expected from the literature.

Several studies have been conducted to assess the role of sex in pain and neuroinflammation [16,44]. Males experience pain differently from females and respond to specific classes of analgesics differently [14]. It has been shown that females have a high prevalence of NP than males [15], possibly because females have a higher prevalence of autoimmune diseases than males and they suffer from many symptoms including NP [45]. The role of astrocytes and microglia in the chronic pain of females and males is still a subject of controversy. Although several studies revealed that microglia contribute to pain states principally in males, other studies propose that microglia might have a role in females [29,46,47,48].

In the current study, naïve female mice were more sensitive to mechanical stimuli than male mice. This finding concurs with previous studies that have shown that female mice are more sensitive than male mice to various stimuli used in pain studies such as mechanical and thermal stimuli [49,50]. This also concurs with studies conducted in humans that show that females are more sensitive to experimentally induced painful stimuli than their male counterparts [51,52]. Moreover, pain is more prevalent in women relative to men and the prevalence of chronic pain has been reported to be higher in women than men [51,52,53].

Previous studies have shown that ddC induces mechanical allodynia in both male and female mice of different strains [9,12,32,33,34,36,54,55,56,57]. In the current study, ddC induced mechanical allodynia to the same degree in both male and female BALB/c mice. These results concur with a recent study that reported that male and female C57BlJ/6 mice developed comparable levels of ddC-induced mechanical allodynia [36]. This is also similar to the study in chemotherapy-induced NP, where no differences in the development of mechanical allodynia were found between male and female mice [58]. These findings with ddC-induced mechanical allodynia are however in contrast to other models of NP, where female mice and rats have been reported to have higher levels of mechanical allodynia than male mice and rats [59,60,61]. This suggests that the presence or lack of sex differences in rodents might depend on the model or type of NP.

In addition to the comparable development of mechanical allodynia, there were sex differences in the gene and protein expression of markers of immune cells and immune-related molecules. Treatment with ddC caused an increase in the mRNA levels of *Cd11b*, *H2-Aa*, *Cd3e*, *Mapk1*, *Il1b*, *Tnf*, and *Il10* transcripts in female, but not male, mice, whereas there were no alterations found in *Gfap* and *Mapk14* transcript levels in both sexes. ddC elevated the levels of CD11b protein and phospho-p38 MAPK in female, but not male, mice, whereas there was an increase in Iba1 protein in male, but not female, mice.

Activation of microglia cells in response to ddC has been implicated in the development of mechanical allodynia as a sign of NP. It has been shown that ddC induced a small increase in OX-42 (CD11b antibody, which is a marker of microglia)-immunoreactivity and an increase in CD11b/c in the spinal dorsal horn of male Wistar rats [35,62]. ddC also increased the immunostaining of Iba1 in the spinal cords of aging male C57BL/BL6 mice [32]. In another study, ddC did not cause any changes in Iba1 staining as a marker of microglia activation in male rats [63]. In this current study, a significant increase in the mRNA expression of microglia markers, *Cd11b,* and *H2-Aa* was detected in the spinal cords of ddC-treated female mice but not in ddC-treated male mice. The levels of CD11b protein were increased in female, but not male, mice, whereas there was an increase in Iba1 protein in male, but not female, mice treated with ddC. This suggests that there is a sex-dependent difference in the ddC-induced upregulation of microglia markers in BALB/c mice. 

Several studies showed that astrocytes are activated in response to antiretroviral drugs. ddC treatment significantly increased the expression of the astrocytes marker GFAP in the spinal cords of male Sprague-Dawley rats [64], male CD-1 mice [57], and male aging C57BL/6 male mice [32]. One study showed a slight rise in the levels of GFAP immunoreactivity in the spinal dorsal horn in male Wistar rats after systemic ddC treatment [35]. However, in the current study, there was no alteration in the relative mRNA and protein expression of GFAP in the spinal cords of both male and female mice treated with ddC similar to what was reported in a previous study, but in the brains of female BALB/c mice [9]. The possible reason for the discrepancy between this study and the previous studies could be due to the different species and strains used. BALB/c mice were used in this study, while in the other studies C57BL/6 mice, CD-1 mice, Wistar rats, and Sprague-Dawley rats were used. 

During neuroinflammation, peripheral T cells are recruited at the site of the injury in the CNS [65]. Various studies suggest that infiltrating T-cells play an important role in mechanical allodynia and hypersensitivity in female mice [30,31]. One study showed a spread of regulatory T cells (Treg) and natural killer (NK) cells in the spinal cords of female mice after intrathecal administration of CSF1 that induced mechanical hypersensitivity [47]. In the current study, ddC induced a significant increase in the transcript levels of *Cd3e*, a T cells marker, in female, but not male, mice, which implies that T cell activation during NP might be important and specific to female mice.

Cytokines are secreted by several immune cells, mainly monocytes (macrophages), lymphocytes, and CNS cells such as neurons, astrocytes, and microglia [66]. In the current study, there was a significant increase in the transcript levels of cytokines (*Tnf*, *Il1b,* and *Il10*) in the spinal cords of female mice at the time they developed ddC-induced mechanical allodynia similar to what has been observed in the brains in a recent study [9]. The source of cytokines in female mice most likely was activated microglia and T cells. In contrast, there was no change in the levels of cytokines (*Tnf*, *Il1b,* and *Il10*) in the spinal cords of ddC-treated male mice. However, our findings contrast with previous studies where there was a significant increase in the protein levels of TNF-α and IL-1ß in the spinal cords of aging C57BL/6 mice [32], and an increase in both gene and protein expression of TNF-α in the spinal cords of male Sprague-Dawley rats [64] after ddC treatment. The differences between our findings in male rodents and previous studies could be due to strain and species differences. Other studies have found differences in the expression of cytokines between mice strains. For example, C57BL/6 had increased *Tnf* and *Il1b* transcripts in the brains compared to BALB/c mice after *C. parvum* antigen injection [67]. While BALB/c had increased IL-1β and TNF-α in the spleen compared to C57BL/6 mice after *B. pseudomallei* infection [68]. Stress was found to induce a Th2 response in BALB/c and a Th1 response in C57BL/6 mice [69] and induced anti-inflammatory cytokine (*Il10*) only in BALB/c mice, but not in C57BL/6 mice [70]. 

The production of cytokines is controlled by several signaling pathways. Some of these critical pathways that have a role in the signal transduction cascade associated with chronic nociception are the mitogen-activated protein kinase (MAPK) pathways [71,72]. In mammalian cells, there are three well-known MAPK pathways such as the ERK1/2, the p38 MAPK α, β, δ, and γ pathways, and the c-Jun N-terminal kinase 1,2, and 3 (c-JNK1/2/3) [73]. The activation of MAPKs in glial cells is fundamental in the development and maintenance of NP [72]. In the current study, there was a significant increase in the relative mRNA expression of *Mapk1* in the spinal cords of ddC-treated female mice. Phosphorylated ERK1/2 was previously shown to be increased in the brains of female BALB/c mice treated with ddC [9], but was not increased in the spinal cords in the current study. On the other hand, levels of phosphorylated p38 MAPK were increased in the spinal cords of female mice treated with ddC, which correlated with microglia activation (increased CD11b expression). The p38 MAPK plays a role in regulating the production and signaling of cytokines such as IL-1β and TNF-α [74,75,76,77]. This might explain why there was an increase in the transcripts of these cytokines in female, but not male, mice because phosphorylated p38 MAPK was increased in female, but not male, mice.

Thus, the current study and various other studies demonstrate that neuroimmune changes are sex-dependent but are also dependent on the strain of mice used as well as the cause of neuropathy and neuroinflammation.

## 5. Conclusions

**Figure 15:**
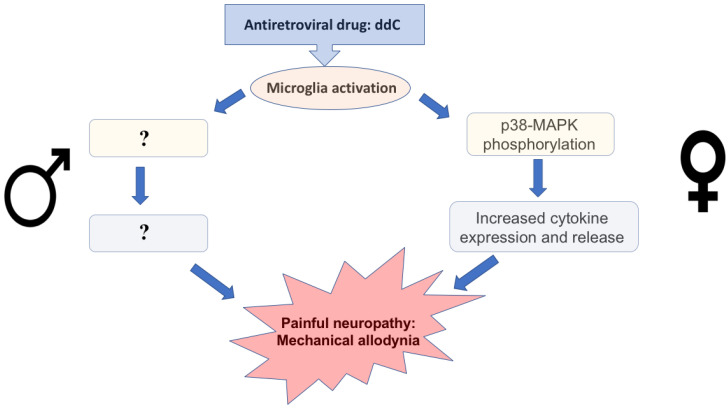
A scheme of the results in the context of the pathophysiology of painful neuropathy. Antiretroviral drugs cause painful peripheral neuropathy in both male and female patients. ddC caused mechanical allodynia, a symptom of NP, in both sexes in mice. Microglia have been reported to play an important role in the pathophysiology of antiretroviral-induced neuropathy. The ddC induced microglia in both sexes of mice. Activated microglia through MAP kinase signaling induces the release of cytokines, which contribute to NP. The ddC induced phosphorylation of p38-MAP kinase and increased the expression of cytokines in female mice but not male mice. This suggests that ddC-induced microglia activation might contribute to NP in male mice via other mechanisms independent of cytokines. This warrants further research.

Figure 15 shows a scheme summarizing the findings of this study in the context of the pathophysiology of painful neuropathy. In conclusion, the current study shows that although female mice were more sensitive to mechanical stimuli than male mice, ddC induced mechanical allodynia to the same extent in both female and male mice. Treatment with ddC increased the transcript levels of microglia markers, T cells, pro-inflammatory and anti-inflammatory cytokines, and *Mapk1* in the spinal cords of female mice. The protein levels of CD11b and phosphorylated p38 MAPK were increased in the spinal cords of female mice treated with ddC, whereas the protein levels of the other microglia marker Iba1 were increased in male mice. There was no change in the mRNA and protein expression of astrocytes marker GFAP in both sexes after treatment with ddC. Therefore, changes in the levels of neuroimmune molecules in the spinal cords of BALB/c mice during antiretroviral drug-induced neuroinflammation are sex-dependent. This suggests that female mice are more prone to antiretroviral drug-induced neuroinflammation than male mice. Further studies are warranted to understand what changes contribute to or are associated with the development of mechanical allodynia in male mice treated with ddC. This could lead to the search for specific therapeutic targets for antiretroviral-induced NP depending on pathogenetic mechanisms.

## Figures and Tables

**Table 1:** Primer sequences of genes whose expression was evaluated by PCR.

Name	Polarity	Sequence 5′to 3′	GenBank *
*Ppia*	Sense	GCTTTTCGCCGCTTGCT	X52803
	Anti-sense	CTCGTCATCGGCCGTGAT	
*Itgam*	Sense	TGCTTACCTGGGTTATGCTTCTG	NM_008401
	Anti-sense	CCGAGGTGCTCCTAAAACCA	
*H2-Aa*	Sense	CTGTGATCAACATCACATGGC	NM_010378
	Anti-sense	TTGTGGAAGGAATAGTCACGG	
*Gfap*	Sense	ACAGCGGCCCTGAGAGAGAT	X02801
	Anti-sense	CTCCTCTGTCTCTTGCATGTTACTG	
*Cd3e*	Sense	TGCTACACACCAGCCTCAAA	NM_007648
	Anti-sense	AGGTCCACCTCCACACAGTA	
*Mapk14*	Sense	AGGCCATGGTGCATGTGTGT	XR-004939533
	Anti-sense	AGTAGCTGGAGGAGGAGGAG	
*Mapk1*	Sense	GCTCACCCTTACCTGGAACA	NM_011952
	Anti-sense	GGACCAGATCCAAAAGGACA	
*Il1b*	Sense	TGGTGTGTACGTTCCCATT	NM_008361
	Anti-sense	CAGCAGAGGCTTTTTTGTTG	
*Tnf*	Sense	GGCTGCCCCGACTACGT	NM_013693
	Anti-sense	GACTTTCTCCTGGTATGAGATAGCAAA	
*Il10*	Sense	CAGCCGGGAAGACAATAACTG	NM_010548
	Anti-sense	CCGCAGCTCTAGGAGCATGT	

* GenBank accession numbers of sequences used for primer design.

## Data Availability

Data will be made available on request.

## References

[1] Croke L. (2019). HIV Prevention and Treatment with ART: International Antiviral Society Updates Recommendations. Am. Fam. Physician.

[2] Arts E.J., Hazuda D.J. (2012). HIV-1 antiretroviral drug therapy. Cold Spring Harb. Perspect. Med..

[3] Panel on Antiretroviral Guidelines for Adults and Adolescents Guidelines for the Use of Antiretroviral Agents in Adults and Adolescents Living with HIV. https://clinicalinfo.hiv.gov/en/guidelines/adult-and-adolescent-arv/what-start-initial-combination-regimens-antiretroviral-naive.

[4] Saag M.S., Gandhi R.T., Hoy J.F., Landovitz R.J., Thompson M.A., Sax P.E., Smith D.M., Benson C.A., Buchbinder S.P., Del Rio C. (2020). Antiretroviral Drugs for Treatment and Prevention of HIV Infection in Adults: 2020 Recommendations of the International Antiviral Society-USA Panel. JAMA.

[5] Badowski M., Perez S.E., Silva D., Lee A. (2020). Two’s a Company, Three’s a Crowd: A Review of Initiating or Switching to a Two-Drug Antiretroviral Regimen in Treatment-Naive and Treatment-Experienced Patients Living with HIV-1. Infect. Dis. Ther..

[6] Arenas-Pinto A., Bhaskaran K., Dunn D., Weller I.V. (2008). The risk of developing peripheral neuropathy induced by nucleoside reverse transcriptase inhibitors decreases over time: Evidence from the Delta trial. Antivir. Ther..

[7] Dalakas M.C. (2001). Peripheral neuropathy and antiretroviral drugs. J. Peripher. Nerv. Syst..

[8] Kallianpur A.R., Hulgan T. (2009). Pharmacogenetics of nucleoside reverse-transcriptase inhibitor-associated peripheral neuropathy. Pharmacogenomics.

[9] Aly E., Khajah M.A., Masocha W. (2019). beta-Caryophyllene, a CB2-Receptor-Selective Phytocannabinoid, Suppresses Mechanical Allodynia in a Mouse Model of Antiretroviral-Induced Neuropathic Pain. Molecules.

[10] Kabba J.A., Xu Y., Christian H., Ruan W., Chenai K., Xiang Y., Zhang L., Saavedra J.M., Pang T. (2018). Microglia: Housekeeper of the Central Nervous System. Cell Mol. Neurobiol..

[11] Joseph E.K., Chen X., Khasar S.G., Levine J.D. (2004). Novel mechanism of enhanced nociception in a model of AIDS therapy-induced painful peripheral neuropathy in the rat. Pain.

[12] Munawar N., Oriowo M.A., Masocha W. (2017). Antihyperalgesic Activities of Endocannabinoids in a Mouse Model of Antiretroviral-Induced Neuropathic Pain. Front. Pharm..

[13] Chen X., Levine J.D. (2007). Mechanically-evoked C-fiber activity in painful alcohol and AIDS therapy neuropathy in the rat. Mol. Pain.

[14] Paller C.J., Campbell C.M., Edwards R.R., Dobs A.S. (2009). Sex-based differences in pain perception and treatment. Pain Med..

[15] Fillingim R.B., King C.D., Ribeiro-Dasilva M.C., Rahim-Williams B., Riley J.L. (2009). Sex, gender, and pain: A review of recent clinical and experimental findings. J. Pain.

[16] Rosen S., Ham B., Mogil J.S. (2017). Sex differences in neuroimmunity and pain. J. Neurosci. Res..

[17] Hanamsagar R., Bilbo S.D. (2016). Sex differences in neurodevelopmental and neurodegenerative disorders: Focus on microglial function and neuroinflammation during development. J. Steroid Biochem. Mol. Biol..

[18] Lenz K.M., Nugent B.M., Haliyur R., McCarthy M.M. (2013). Microglia are essential to masculinization of brain and behavior. J. Neurosci..

[19] VanRyzin J.W., Pickett L.A., McCarthy M.M. (2018). Microglia: Driving critical periods and sexual differentiation of the brain. Dev. Neurobiol..

[20] Han J., Fan Y., Zhou K., Blomgren K., Harris R.A. (2021). Uncovering sex differences of rodent microglia. J. Neuroinflammation.

[21] Green T., Flash S., Reiss A.L. (2019). Sex differences in psychiatric disorders: What we can learn from sex chromosome aneuploidies. Neuropsychopharmacology.

[22] Gold S.M., Willing A., Leypoldt F., Paul F., Friese M.A. (2019). Sex differences in autoimmune disorders of the central nervous system. Semin. Immunopathol..

[23] Zagni E., Simoni L., Colombo D. (2016). Sex and Gender Differences in Central Nervous System-Related Disorders. Neurosci. J..

[24] Guneykaya D., Ivanov A., Hernandez D.P., Haage V., Wojtas B., Meyer N., Maricos M., Jordan P., Buonfiglioli A., Gielniewski B. (2018). Transcriptional and Translational Differences of Microglia from Male and Female Brains. Cell Rep..

[25] Lenz K.M., McCarthy M.M. (2015). A starring role for microglia in brain sex differences. Neuroscientist.

[26] Beggs S., Trang T., Salter M.W. (2012). P2X4R+ microglia drive neuropathic pain. Nat. Neurosci..

[27] Coull J.A., Beggs S., Boudreau D., Boivin D., Tsuda M., Inoue K., Gravel C., Salter M.W., De Koninck Y. (2005). BDNF from microglia causes the shift in neuronal anion gradient underlying neuropathic pain. Nature.

[28] Tsuda M., Shigemoto-Mogami Y., Koizumi S., Mizokoshi A., Kohsaka S., Salter M.W., Inoue K. (2003). P2X4 receptors induced in spinal microglia gate tactile allodynia after nerve injury. Nature.

[29] Sorge R.E., Mapplebeck J.C., Rosen S., Beggs S., Taves S., Alexander J.K., Martin L.J., Austin J.S., Sotocinal S.G., Chen D. (2015). Different immune cells mediate mechanical pain hypersensitivity in male and female mice. Nat. Neurosci..

[30] Cao L., DeLeo J.A. (2008). CNS-infiltrating CD4+ T lymphocytes contribute to murine spinal nerve transection-induced neuropathic pain. Eur. J. Immunol..

[31] Costigan M., Moss A., Latremoliere A., Johnston C., Verma-Gandhu M., Herbert T.A., Barrett L., Brenner G.J., Vardeh D., Woolf C.J. (2009). T-cell infiltration and signaling in the adult dorsal spinal cord is a major contributor to neuropathic pain-like hypersensitivity. J. Neurosci..

[32] Yuan S., Shi Y., Guo K., Tang S.J. (2018). Nucleoside Reverse Transcriptase Inhibitors (NRTIs) Induce Pathological Pain through Wnt5a-Mediated Neuroinflammation in Aging Mice. J. Neuroimmune Pharm..

[33] Masocha W., Thomas A. (2019). Indomethacin plus minocycline coadministration relieves chemotherapy and antiretroviral drug-induced neuropathic pain in a cannabinoid receptors-dependent manner. J. Pharm. Sci..

[34] Sanna M.D., Quattrone A., Ghelardini C., Galeotti N. (2014). PKC-mediated HuD-GAP43 pathway activation in a mouse model of antiretroviral painful neuropathy. Pharm. Res..

[35] Wallace V.C., Blackbeard J., Segerdahl A.R., Hasnie F., Pheby T., McMahon S.B., Rice A.S. (2007). Characterization of rodent models of HIV-gp120 and anti-retroviral-associated neuropathic pain. Brain.

[36] Carey L.M., Xu Z., Rajic G., Makriyannis A., Romero J., Hillard C., Mackie K., Hohmann A.G. (2023). Peripheral sensory neuron CB2 cannabinoid receptors are necessary for both CB2-mediated antinociceptive efficacy and sparing of morphine tolerance in a mouse model of anti-retroviral toxic neuropathy. Pharm. Res..

[37] Richner M., Jager S.B., Siupka P., Vaegter C.B. (2017). Hydraulic Extrusion of the Spinal Cord and Isolation of Dorsal Root Ganglia in Rodents. J. Vis. Exp..

[38] Masocha W. (2009). Systemic lipopolysaccharide (LPS)-induced microglial activation results in different temporal reduction of CD200 and CD200 receptor gene expression in the brain. J. NeuroImmunol..

[39] Amin D.N., Vodnala S.K., Masocha W., Sun B., Kristensson K., Rottenberg M.E. (2012). Distinct Toll-like receptor signals regulate cerebral parasite load and interferon alpha/beta and tumor necrosis factor alpha-dependent T-cell infiltration in the brains of Trypanosoma brucei-infected mice. J. Infect. Dis..

[40] Masocha W., Amin D.N., Kristensson K., Rottenberg M.E. (2008). Differential invasion of Trypanosoma brucei brucei and lymphocytes into the brain of C57BL/6 and 129Sv/Ev mice. Scand. J. Immunol..

[41] Masocha W., Rottenberg M.E., Kristensson K. (2006). Minocycline impedes African trypanosome invasion of the brain in a murine model. Antimicrob. Agents ChemoTher..

[42] Mohamed M.Y., Masocha W. (2020). Indomethacin augments lipopolysaccharide-induced expression of inflammatory molecules in the mouse brain. PeerJ.

[43] Livak K.J., Schmittgen T.D. (2001). Analysis of relative gene expression data using real-time quantitative PCR and the 2(-Delta Delta C(T)) Method. Methods.

[44] Keogh E. (2008). Sex Differences in Pain. Rev. Pain.

[45] Campbell J.N., Meyer R.A. (2006). Mechanisms of neuropathic pain. Neuron.

[46] Kisucka A., Bimbova K., Bacova M., Galik J., Lukacova N. (2021). Activation of Neuroprotective Microglia and Astrocytes at the Lesion Site and in the Adjacent Segments Is Crucial for Spontaneous Locomotor Recovery after Spinal Cord Injury. Cells.

[47] Kuhn J.A., Vainchtein I.D., Braz J., Hamel K., Bernstein M., Craik V., Dahlgren M.W., Ortiz-Carpena J., Molofsky A.B., Molofsky A.V. (2021). Regulatory T-cells inhibit microglia-induced pain hypersensitivity in female mice. Elife.

[48] Nieto F.R., Clark A.K., Grist J., Hathway G.J., Chapman V., Malcangio M. (2016). Neuron-immune mechanisms contribute to pain in early stages of arthritis. J. Neuroinflammation.

[49] Barrett A.C., Smith E.S., Picker M.J. (2002). Sex-related differences in mechanical nociception and antinociception produced by mu- and kappa-opioid receptor agonists in rats. Eur. J. Pharm..

[50] Parvathy S.S., Masocha W. (2015). Coadministration of indomethacin and minocycline attenuates established paclitaxel-induced neuropathic thermal hyperalgesia: Involvement of cannabinoid CB1 receptors. Sci. Rep..

[51] Bartley E.J., Fillingim R.B. (2013). Sex differences in pain: A brief review of clinical and experimental findings. Br. J. Anaesth.

[52] Sorge R.E., Strath L.J. (2018). Sex differences in pain responses. Curr. Opin. Physiol..

[53] Sorge R.E., Totsch S.K. (2017). Sex Differences in Pain. J. Neurosci. Res..

[54] Sanna M.D., Quattrone A., Mello T., Ghelardini C., Galeotti N. (2014). The RNA-binding protein HuD promotes spinal GAP43 overexpression in antiretroviral-induced neuropathy. Exp. Neurol..

[55] Sanna M.D., Peroni D., Quattrone A., Ghelardini C., Galeotti N. (2015). Spinal RyR2 pathway regulated by the RNA-binding protein HuD induces pain hypersensitivity in antiretroviral neuropathy. Exp. Neurol..

[56] Sanna M.D., Ghelardini C., Galeotti N. (2016). Blockade of the spinal BDNF-activated JNK pathway prevents the development of antiretroviral-induced neuropathic pain. Neuropharmacology.

[57] Sanna M.D., Ghelardini C., Galeotti N. (2017). Spinal astrocytic c-Jun N-terminal kinase (JNK) activation as counteracting mechanism to the amitriptyline analgesic efficacy in painful peripheral neuropathies. Eur. J. Pharm..

[58] Naji-Esfahani H., Vaseghi G., Safaeian L., Pilehvarian A.A., Abed A., Rafieian-Kopaei M. (2016). Gender differences in a mouse model of chemotherapy-induced neuropathic pain. Lab. Anim..

[59] Ahlstrom F.H.G., Matlik K., Viisanen H., Blomqvist K.J., Liu X., Lilius T.O., Sidorova Y., Kalso E.A., Rauhala P.V. (2021). Spared Nerve Injury Causes Sexually Dimorphic Mechanical Allodynia and Differential Gene Expression in Spinal Cords and Dorsal Root Ganglia in Rats. Mol. Neurobiol..

[60] Lynch J.L., Alley J.F., Wellman L., Beitz A.J. (2008). Decreased spinal cord opioid receptor mRNA expression and antinociception in a Theiler’s murine encephalomyelitis virus model of multiple sclerosis. Brain Res..

[61] Rahn E.J., Iannitti T., Donahue R.R., Taylor B.K. (2014). Sex differences in a mouse model of multiple sclerosis: Neuropathic pain behavior in females but not males and protection from neurological deficits during proestrus. Biol. Sex Differ..

[62] Blackbeard J., Wallace V.C., O’Dea K.P., Hasnie F., Segerdahl A., Pheby T., Field M.J., Takata M., Rice A.S. (2012). The correlation between pain-related behaviour and spinal microgliosis in four distinct models of peripheral neuropathy. Eur. J. Pain.

[63] Zheng F.Y., Xiao W.H., Bennett G.J. (2011). The response of spinal microglia to chemotherapy-evoked painful peripheral neuropathies is distinct from that evoked by traumatic nerve injuries. Neuroscience.

[64] Zheng X., Ouyang H., Liu S., Mata M., Fink D.J., Hao S. (2011). TNFalpha is involved in neuropathic pain induced by nucleoside reverse transcriptase inhibitor in rats. Brain Behav. Immun.

[65] Yang Q., Wang G., Zhang F. (2020). Role of Peripheral Immune Cells-Mediated Inflammation on the Process of Neurodegenerative Diseases. Front. Immunol..

[66] Jeon S.W., Kim Y.K. (2016). Neuroinflammation and cytokine abnormality in major depression: Cause or consequence in that illness?. World J. Psychiatry.

[67] Sheng W.S., Hu S., Lamkin A., Peterson P.K., Chao C.C. (1996). Susceptibility to immunologically mediated fatigue in C57BL/6 versus Balb/c mice. Clin. Immunol. Immunopathol..

[68] Koo G.C., Gan Y.H. (2006). The innate interferon gamma response of BALB/c and C57BL/6 mice to in vitro Burkholderia pseudomallei infection. BMC Immunol..

[69] Palumbo M.L., Canzobre M.C., Pascuan C.G., Rios H., Wald M., Genaro A.M. (2010). Stress induced cognitive deficit is differentially modulated in BALB/c and C57Bl/6 mice: Correlation with Th1/Th2 balance after stress exposure. J. NeuroImmunol..

[70] Sathyanesan M., Haiar J.M., Watt M.J., Newton S.S. (2017). Restraint stress differentially regulates inflammation and glutamate receptor gene expression in the hippocampus of C57BL/6 and BALB/c mice. Stress.

[71] Ji R.R. (2004). Peripheral and central mechanisms of inflammatory pain, with emphasis on MAP kinases. Curr. Drug Targets Inflamm. Allergy.

[72] Ji R.R., Gereau R.W.T., Malcangio M., Strichartz G.R. (2009). MAP kinase and pain. Brain Res. Rev..

[73] Raman M., Chen W., Cobb M.H. (2007). Differential regulation and properties of MAPKs. Oncogene.

[74] Bachstetter A.D., Van Eldik L.J. (2010). The p38 MAP Kinase Family as Regulators of Proinflammatory Cytokine Production in Degenerative Diseases of the CNS. Aging Dis..

[75] Berta T., Park C.K., Xu Z.Z., Xie R.G., Liu T., Lu N., Liu Y.C., Ji R.R. (2014). Extracellular caspase-6 drives murine inflammatory pain via microglial TNF-alpha secretion. J. Clin. Investig..

[76] Clark A.K., D’Aquisto F., Gentry C., Marchand F., McMahon S.B., Malcangio M. (2006). Rapid co-release of interleukin 1beta and caspase 1 in spinal cord inflammation. J. Neurochem..

[77] Ji R.R., Suter M.R. (2007). p38 MAPK, microglial signaling, and neuropathic pain. Mol. Pain.

